# Maintaining physical activity following myocardial infarction: a qualitative study

**DOI:** 10.1186/s12872-021-01898-7

**Published:** 2021-02-18

**Authors:** Alex Coull, Gemma Pugh

**Affiliations:** Centre for Sports and Exercise Medicine, Queen Mary University of London, Mile End Hospital, Bancroft Road, London, E1 4DG UK

**Keywords:** Physical activity, Myocardial infarction, Qualitative study, Cardiac rehabilitation, Behaviour change

## Abstract

**Background:**

Outcomes following myocardial infarction (MI) are improved by uptake and maintenance of physical activity (PA), but little is understood regarding patients experience of maintaining an active lifestyle once immediate support, such as cardiac-rehabilitation (CR), has ended.

**Aim:**

The purpose of this study was to investigate MI survivors’ attitude and appraisal towards PA and the perceived barriers, motivators and facilitators for maintaining PA long-term.

**Methods:**

Semi-structured interviews were carried out with 18 adults (mean age 60.5, range 37–73 years) from England and Scotland, who were a minimum of 5 months post-MI (mean 29 months, range 5–122 months). There were comparatively more male participants (n = 13, 72 %) than female (n = 5, 28 %). Overall 12 (67 %) participants had attended CR. The interviews were transcribed verbatim and thematic analysis was performed using qualitative data analysis software NVivo.

**Results:**

Data analysis indicated that the following four core themes influenced MI survivors’ behaviour and attitude towards PA: (1) MI as a teachable moment for behaviour change, (2) affective response to MI: enjoyment versus fear, (3) cognitive response to MI: self-perception, attitude and self-efficacy, and (4) access to support and resources, including PA facilities and social support. Participants highlighted a lack of available guidance on maintaining PA behaviour change following CR and that advice on the frequency and intensity of exercise to follow was often unclear and confusing. Feelings of vulnerability and fear of overexertion were apparent, affecting participants self-efficacy to exercise.

**Conclusions:**

Current CR programmes fail to address PA belief systems and perceptions of self-efficacy to exercise. Interventions that address feelings of vulnerability and fear of overexertion may be beneficial. Providing ongoing PA advice and access to social support may facilitate patients to maintain changes in PA.

**Supplementary Information:**

The online version contains supplementary material available at 10.1186/s12872-021-01898-7.

## Introduction

Myocardial Infarction (MI) is a leading cause of premature mortality and morbidity worldwide. It places a significant burden on healthcare systems. At present, there are an estimated 1 million MI-survivors living in the UK [1]. Improved survival after MI has been attributed to secondary prevention measures that include cardiac-rehabilitation (CR) programmes [2]. Based upon strong evidence that physical activity (PA) reduces cardiovascular mortality and improves patient quality of life [3], structured PA is a core element of the lifestyle management component of CR programmes [4, 5]. Randomised controlled-trials (RCTs) demonstrate that CR programmes lead to behaviour change and a significant improvement in PA levels among patients with cardiovascular disorders [6]. However, once CR support ends many patients fail to maintain the moderate levels of PA required for stable cardiovascular health. An estimated 46.6 % of MI survivors do not meet PA recommendations 1 year after rehabilitation [7].

Existing studies investigating determinants of PA indicate socioeconomic status, weight status, presence of co-morbidities and low exercise capacity account for variance in PA behaviour among CR attendees and those with high cardiovascular risk [7, 8]. Qualitative studies suggest psychosocial factors including beliefs, knowledge, access to facilitates and social support also influence the PA levels of individuals with cardiovascular disease [9]. However, most of these studies investigate PA behaviour change among individuals diagnosed with cardiovascular risk factors (hypertension, hyperlipidaemia, metabolic syndrome or type II diabetes), or individuals who have recently had a MI and are, at the time of the study, participating in formal CR programmes. As a result, there is limited understanding of the factors influencing PA behaviour among MI patients who have been living with their condition over the long term and are no longer eligible for formal support. This is an important and relevant group to study, as MI is known to be a life-changing event which prompts an individual to re-evaluate their lifestyle and health choices.

Moreover, despite being central to many theoretical behavioural models of behaviour change [10], little is understood regarding cognitive (e.g. knowledge and understanding) and affective (e.g. attitude and appraisal) factors which may determine maintenance of PA behaviour among MI survivors. Exploration of these elements of life post-MI promotes a more contextualised and empathic understanding of PA in this population [11]. This is particularly relevant to primary care physicians responsible for cardiovascular disease care and will aid the development more tailored interventions designed to help patients sustain changes in their PA behaviours.

Therefore, the purpose of this study was to explore factors influencing MI survivors PA behaviour. Specifically, this study aimed to investigate MI survivors’ attitude and appraisal towards PA and the perceived barriers, motivators and facilitators for maintaining PA long-term.

## Methods

### Design

This qualitative study was designed using grounded theory methodology, first introduced by Glaser and Strauss in 1967 [12]. Data were collected via semi-structured, one-to-one telephone interviews conducted by the same researcher and recorded by voice recorder. The interview topic guide (Additional file 1: A) was designed to encourage participants to speak freely about their experience of PA following MI, so was constructed of open questions, the order of which was not strictly adhered to. Questions covered day-to-day lifestyle post MI, current levels of PA, motivation to be active, knowledge of the benefit of PA among MI patients, barriers to being active and their experience of seeking PA support post-CR. Prior to the interview, participants were asked to complete a short-questionnaire on their demographic (age, gender, occupation status) and health details (hearing, memory and other physical ailments). This information was used to deem whether they were medically fit for interview, which all interested participants were. Written field notes were taken post-interview that included the researcher’s initial thoughts and impressions. Interviews were limited to 40 minutes, except where new insights were still emerging that the participant was willing to discuss further. Data collection continued until it was felt the sample was representative of the post-MI demographic and theoretical saturation was reached [13]. The investigation conforms with the principles outlined in the Declaration of Helsinki. Ethical approval for this study was granted by the QMUL Ethics of Research Committee Approval Number QMREC2018/48/012.

### Sample recruitment

MI survivors were recruited via charity health forums, cardiac support groups and gymnasiums. Participant inclusion and exclusion criteria are detailed in Table 1. If an individual was eligible and interested in taking part in the study, they were asked to familiarise themselves with the study information sheet and provide informed written consent before completing a pre-interview questionnaire. They were then asked to arrange a telephone interview with a member of the research team at a convenient time. Due to the nature of recruitment, we were unable to obtain medical records in order to confirm participants’ diagnosis of MI, instead this was verbally confirmed by each participant at the start of interview, along with the date it/they had occurred and location where they received care. Of the 20 people who made initial contact with the authors (having seen the study advert), 2 did not respond to further emails from the team on 3 or more occasions thereafter and hence did not participate in the interviews.

**Table 1:** Participant selection criteria

Inclusion criteria	Exclusion criteria
Adults (minimum 18 years old)	< 18 years old
Previous diagnosis of MI by a physician*	MI not confirmed by physician and/or chest pain attributed to another cause
MI occurred > 5 months pre-interview	MI within previous 5 months
English language understood and spoken fluently	Cannot converse fluently in English language
Permanent address in the UK	Living predominantly outside the UK
Medically fit to undertake interview	Hearing and/or memory impairment including dementia severe enough to affect ability to accurately answer questions and recall information

*As per the World Health Organization (WHO) criteria for diagnosing MI: at least 2 of the following 3 criteria were required: typical chest pain, elevated serum cardiac enzymes, ischaemic ECG changes (20)

### Analysis

Data were analysed using the 6-step process of thematic analysis outlined by Braun and Clarke [14]. Recordings were transcribed verbatim and checked for accuracy during the initial familiarisation phase. NVivo qualitative data analysis software version 12 (QSR International Pty Ltd) was used to code text and search for initial themes. These themes were discussed with a second researcher and cross-checked within the transcripts before being defined into an indexing scheme. The transcripts were then re-analysed using these themes contained within the indexing scheme. The researchers then discussed and synthesized the themes resulting in the final themes reported within this paper. The COnsolidated criteria for REporting Qualitative research (COREQ) was followed, please refer to Additional file 1. 

## Results

A total of eighteen post-MI patients (mean age: 60.5 years, range 37–73 years) took part in the study. Table 2 displays participant characteristics. Participants were predominantly male (n = 13, 72 %), employed (n = 10, 56 %) and > 2 years post MI (mean 29 months; range 5**–**122 months). Mean age at MI was 58.2 years (range 37–73 years). Participants came from a range of locations across England and Scotland and pre-morbid PA status varied from inactive to very fit. Most (n = 17, 94 %) had suffered a single MI and a majority (n = 12, 67 %) had attended CR.

**Table 2:** Participant characteristics

Gender	Age at MI (years)	Time since MI (months)	Region of UK (location of treatment and follow-up)	CR attendance	Work status	PA level pre-MI (see guide below)	PA level post-MI (see guide below)
M	61	7	South West	Y	Part-time	2	3
F	42	37	West Midlands	Y	Part-time	3	4
M	60	8	Greater London	N	Retired	5	4
M	60	122*	South East	N	Retired	4	4
F	61	18	North East	Y	Retired	2	3
M	63	5	Scotland	Y	Full-time	3	3
F	70	7	North West	N	Retired	2	1
F	58	13	Scotland	N	Part-time	3	2
M	48	15	East of England	N	Full-time	4	5
M	66	23	Yorkshire and the Humber	Y	Retired	4	3
M	63	28	Greater London	Y	Part-time	2	4
M	69	12	South West	Y	Retired	3	3
M	41	12	West Midlands	Y	Full-time	1	3
M	73	74	South West	Y	Retired	4	3
M	58	82	Greater London	Y	Retired	3	4
M	56	23	South East	Y	Full-time	3	3
F	37	12	North East	Y	Full-time	2	4
M	62	29	Greater London	N	Full-time	5	5

Physical Activity Guide

1 = Usually inactive. Minimal daily activity. Spends most of the day sitting down. Would opt to use transport such as a car even for short journeys. May be wheelchair bound

2 = Low intensity activity on most days e.g. walking at a leisurely pace, light housework. No strenuous activity; rarely has to stop to catch their breath

3 = Moderate activity such as yoga or swimming 1–2 times per week. Generally active at other times e.g. walks the dog, plays with children/grandchildren. Job may have physical demands e.g. a maintenance worker, delivery driver or carer

4 = High intensity. PA on 3 or more days a week such as circuits classes, rackets sports, or jogging/running. Consciously active throughout the day with reduced sitting time. May have an active commute such as cycling

5 = High fitness level. Consistently trains to a high intensity or for prolonged durations. Works to increase fitness goals. Pushes self to complete challenges and events e.g. half marathon or triathlon

M, male; F, female; Y, yes; N, no; CR, cardiac rehabilitation; PA, physical activity

*More than 1 MI

Individual interviews typically lasted 40 minutes in duration (range, 28–66 min). Four core themes emerged which appeared to influence heart attack survivors PA behaviour: (1) MI as a teachable moment for behaviour change; (2) affective emotional response to MI: enjoyment versus fear; (3) cognitive response to MI: self-perception, attitude and self-efficacy; and (4) access to support and resources, including PA facilities and social support.

### Theme 1: MI as a teachable moment for PA behaviour change

Suffering from MI was regarded as a *“life-changing”* event that motivated many to eliminate destructive health behaviours and focus on making the *“right choices”* in the future. Although many participants were broadly aware that PA was beneficial to health, it took them suffering a MI to fully appreciate the stark risks of inactivity including greater risk of another cardiac event. Participants often discussed that their MI had come as a “wake-up call” that forced them to consider their previous habits and lifestyle choices. The motivation to improve physical fitness immediately following MI was particularly evident among those with low pre-morbid PA levels.

The heart attack made me wake up a bit and think actually I need to get fitter; I need to do stuff now and stay healthy otherwise I’m going to end up having another one or even worse.

Male; time since MI, 7 months; PA level, 2 pre-MI to 3 post-MI

It’s like my body failed itself at 40… it spurred me get me through rehab. I’ve got to get healthier; I’m going to be healthier than what I was.

Female; time since MI, 37 months; PA level, 3 pre-MI to 4 post-MI

Several participants remarked the ease with which they had achieved considerable weight loss through PA and diet in a short space of time. However, one participant noted that although MI had served as a powerful instigator for her to quit smoking and become more active, she now found herself relapsing to poor habits. She implied the impact of the MI as a shock to change behaviour had somewhat diminished with time, as other life stressors became more prominent, leading to a relapse of poor habits such as physical inactivity that acted as coping strategies.

I lay there the first morning thinking, well, there’s nothing whatsoever I can do about the physical situation, whatever damage has happened, has happened, but I’m here, and I made a very, very conscious decision that I would do the right things.

Male; time since MI, 5 months; PA level, 3 pre-MI to 3 post-MI

### Theme 2: Affective emotional response to MI and PA: enjoyment versus fear

Many participants described PA as a positive escape. One participant described how running created a *“happy place”* and another described how yoga *“calmed the brain and focused the mind”.* Several participants identified exercise as a solution to their negative thought spirals relating to their MI.

Some days I’ve convinced myself that I’m having another heart attack and that today is my last day… I can’t pin down the thing that sparks the negative thoughts that sets that off, but I can pin down the exercise being the thing that kind of zaps you back out of it.

Male; time since MI, 12 months; PA level, 1 pre-MI to 3 post-MI

However, despite most participants describing joy from remaining active, many also described feelings of fear and anxiety over their cardiovascular health. Following the *“complete shock”* of MI, many reported low mood and depressive thoughts. One participant described facing *“a really black abyss some days”*, which made PA challenging. Several were fearful of leaving the house post-MI; *“you don’t know what your body can do”.* Fear of exercising alone and/or fear of being far from help, such as by going on a solo rural jog versus a doing a supervised gym class, created further barriers for some.

I don’t feel confident enough to try doing it on my own in case I do too much. Even an angina attack would panic me and then you start having a panic attack and your heart rate goes up and you think you are having another heart attack.

Female; time since MI, 7 months; PA level, 2 pre-MI to 1 post-MI

Chest pain that occurred during PA and at other times was a commonly reported symptom that caused apprehension of a further cardiac event, increased anxiety and often led to A&E attendance. Participants had become vigilant of warning signs; *“if it went into my jaw and into my arm, then I would really start worrying”*. The fear of an exercise induced cardiac event was evident, despite the participant’s previous activity level or perceived self-efficacy to exercise.

Many participants believed monitoring of HR during PA was important. One self-described *“hyper-cautionary”* participant found that despite feeling completely well, he only felt reassured that no further cardiac damage was occurring if he monitored his HR meticulously.

Everybody that hasn’t been a consultant has basically said, ‘Your body will tell you’, but I keep using the two criteria, my body will tell me but what’s my pulse rate? And I’ve always been a hyper-cautionary person on every single thing in my life, so I’m not going to take a risk especially with something where the outcome can be so fatal.

Male; time since MI, 23 months; PA level, 4 pre-MI to 3 post-MI

Side effects of new medications prescribed as secondary prevention for further MI limited some participants’ ability to exercise; for example, fatigue caused by beta blockers or statin-induced leg cramps. The side effects of the MI itself were a limiting factor for others.

The beta blockers they got me on, they screw me up a little bit. I don’t have a great deal of energy, so I always run first thing in the morning before they kick in.

Male; time since MI, 15 months; PA level, 4 pre-MI to 5 post-MI

The aftereffect of the heart attack meant that I was very short of breath. So, it’s quite a struggle for me to walk anywhere.

Male; time since MI, 74 months; PA level, 4 pre-MI to 3 post-MI

Several co-morbidities including fibromyalgia and mechanical back pain reoccurred among participants and negatively impacted their ability to perform and/or enjoy PA.

Walking for me, because of these long, long term spinal problems, is extremely difficult, and I haven’t walked more than really to the end of our drive in months.

Female; time since MI, 7 months; PA level, 2 pre-MI to 1 post-MI

### Theme 3: The cognitive response to MI and PA: self‐perception, attitude, and self‐efficacy

It was evident that participants’ attitude towards exercise based rehabilitation and self-efficacy to exercise influenced their physical activity behaviour post MI and CR. One participant felt that by opting for a healthier lifestyle that included more PA he had become *“a totally different person”*. Contrastingly, another felt *“frustrated a lot of the time”* in regard to his apparent identity change; he had been *“regarded as this really healthy, fit person”* prior to the MI, but found that *“suddenly it had all changed”*. Generally, those who had the self-perception they were a *“fit and active”* person found it easier to return to their high pre-morbid PA level.

I am fit, I’ve always been fit, and to some degree they say that that’s the reason I’m still here… I’ve always been active, and I guess it’s in my psyche that I want to be active. I don’t want to be sitting around doing nothing.

Male; time since MI, 12 months; PA level, 3 pre-MI to 3 post-MI

A *“just get on with it”* attitude relating to exercising post-MI was heard by some. One participant described themselves as a *“positive person”*, while another *“carried on like nothing had happened”*, despite experiencing exertional chest pain.

In the back of my mind I know that this is probably what’s going to kill me at the end of the day, but you’ve got to enjoy life when it’s there and now I enjoy life, just chucking on my shoes and getting out there running.

Male; time since MI, 15 months; PA level, 4 pre-MI to 5 post-MI

Some participants found setting goals for progression and maintenance of positive PA behaviours over the longer term helpful and worked towards attaining incremental milestones. Some with co-morbidities felt frustrated at being unable to *“get back”* to pre-MI PA levels and had to adjust their goals and expectations. Self-monitoring techniques such as using devices to record step count or HR were common. Some used these in a constructive and motivational way to achieve their goals, others found them detrimental and restricting.

My exercise regime has changed considerably to the point where basically I bought myself a [activity tracker] and I tell people that the [activity tracker] saved my life… My target is 15,000, 16,000 steps a day

Male; time since MI, 12 months; PA level, 1 pre-MI to 3 post-MI

I monitor my heart rate and everything quite closely, almost obsessively so. So, I’ve tried to step back a bit from that because I noticed that I was waking up in the middle of the night and checking my heart rate

Female; time since MI, 18 months; PA level, 2 pre-MI to 3 post-MI

### Theme 4: Access to support and resources: including exercise facilities and social support

Most participants described excellent experiences of CR, praising the supported and controlled nature of the exercise classes and relevance of the educational sessions. Despite the positive feedback regarding CR support it was apparent that there is a lack of tailored PA advice suited to individual needs. For example, one participant who had high pre-MI levels PA described declining CR on the basis that he felt the exercise classes weren’t suitable or necessary for those who were already comfortable exercising. However, in his case, more specific advice on how to safely build up his training capacity and intensity would have been greatly valued. Additionally, by declining CR such participants also missed out on the psychological support and educational sessions.

The vast majority of people who have myocardial infarction are people who are considerably unfit… so the class has to be really, really basic and quite rightly so, but that would have been absolutely no use to me whatsoever.

Male; time since MI, 29 months; PA level, 5 pre-MI to 5 post-MI

A predominantly wheelchair bound participant expressed frustration at the lack of CR services available to those with physical disabilities. She also felt there was a lack of training among gym staff as to how to support her to be active safety, outsides the constraints of a group exercise class.

I asked about it and she said, “Ooh, you are on a mobility scooter, I don’t think you’ll be able to do it”. After that, I didn’t see her again at all.

Female; time since MI, 7 months; PA level, 2 pre-MI to 1 post-MI

Participants’ description of the availability of follow-on exercise programmes post-CR varied widely from well-established community groups to no continued support. A lack of general health promotion guidance and the perception that there was *“no one to tell you what to do”* commonly created a feeling of vulnerability and loss of confidence among participants. Participants often described how lack of support and guidance made the maintenance of positive PA behaviours more challenging.

You come out of cardio rehab and it’s just like you’ve got to drop straight into the ocean, because there’s nothing there. You go to the gym and you go, right, I’ve gone through this, and they look at you, blank. Unless they’ve got a specialist cardio rehab programme.

Female; time since MI, 37 months; PA level, 3 pre-MI to 4 post-MI

One of the circuits is a carry-on from the cardiac rehab and it’s run by the local leisure centre, so some of the people who run those circuits have run the cardiac rehab in the past… they have picked up that there’s a gap between people finishing cardiac rehab and then going on to other things, so they’ve provided extra circuits for people.

Female; time since MI, 18 months; PA level, 2 pre-MI to 3 post-MI

Social support from family and friends emerged as a powerful facilitator of PA. Many participants disclosed that since their original MI their family was supporting them to make physical activity a regular habit.

My wife is my motivation; she nags me into it

Male; time since MI, 23 months; PA level, 3 pre-MI to 3 post-MI

My son has just started going to the gym and he’s 14 and he comes with me and it’s really nice because we have a chat when we’re working out and he will tell me more at the gym than when he comes in from school

Female; time since MI, 12 months; PA level, 2 pre-MI to 4 post-MI

Online resources, particularly the British Heart Foundation (BHF) forum, were highly praised as they allowed participants share PA stories and seek reassurance from other MI survivors. Some participants started volunteering for the BHF to *“give something back”* by passing on their knowledge and experience. Those who had utilised their GP reported them to have been *“outstanding”* and *“fabulous”*, someone they can *“talk to sensibly to”* about PA issues. Participants generally disliked that there was no routine long-term contact with *“the specialist”*. Many felt uneasy having been *“signed off”* by the cardiologists and expressed they would *“like more reassurance that everything is working properly”* potentially via an annual follow-up appointment, rather than only being able to see them in an emergency when it has *“all gone wrong”.*

I’d like to think that, say, a year after the procedure that somebody wants to have a look at you and say, “Well yes, it’s all okay, that’s fine, come back and see me in a years’ time”. Because my wife has a pacemaker and she is checked every year and it seems illogical that you don’t check somebody who has had a major procedure.

Male; time since MI, 12 months; PA level, 3 pre-MI to 3 post-MI

## Discussion

The purpose of this study was to explore factors influencing MI survivors PA behaviour. Four core themes emerged: (1) MI as a teachable moment for behaviour change; (2) affective emotional response to MI: enjoyment vs. fear; (3) cognitive response to MI: self-perception, attitude and self-efficacy; and (4) access to support and resources. It was clear that internal psychological factors including fear were related to self-efficacy to exercise and that advice on the frequency and intensity of exercise to follow post-MI was often unclear and confusing. Interventions which improve MI survivors’ self-efficacy to exercise by addressing health beliefs and perceived ability to be active may overcome low levels of PA maintenance [15]. Moreover, it was evident that providing ongoing PA advice and access to social support may facilitate MI survivors to maintain changes in PA acquired during CR programmes.

A MI is a life-changing changing event that often prompts individuals to change their behaviour (e.g. increase PA, quit smoking, lose weight) as a means to reduce cardiovascular complications and risk of mortality [16]. Hence MI can be described as a teachable moment, acknowledged in conceptual models of behaviour, as a naturally occurring health event that motivates a person to adopt risk-reducing behaviours [17–19]. Data from this study confirm this observation and suggest healthcare professionals can utilise the patient’s emotional response following MI and their new perception of personal risk to encourage positive PA habits [17]. Similarly, motivation to make lifestyle changes gained through public health programmes such as CR can be complemented and reinforced by individualised guidance from primary care physicians responsible for chronic cardiovascular disease care [20]. Primary care physicians are well placed to also promote lifestyle behaviour change as a proactive means for MI patients to regain control over their health status and medical condition [21].

This study found affective emotional responses, including concern, fear and anxiety, dramatically influenced PA behaviour post-MI. A ‘*fear of dying’*, a ‘*fear of PA’* and a ‘*fear of dying whilst exercising’* were common. Professionally led, exercise-based CR can overcome these thought patterns commonly observed among patients post-MI. For example, a Swedish based study found attending exercise-based rehabilitation programmes significantly reduced fear-avoidance beliefs highlighting the benefit of CR for MI patients worried or anxious about overexertion [22]. More generally, PA is consistently associated with better psychological functioning and quality of life among MI survivors [23]. A recent meta-analysis found exercise-based CR had a significant effect in reducing post-MI anxiety and depression, which played an important role in enhancing quality of life [24]. Overcoming the anxiety related to PA post-MI is essential. Providing MI survivors with exercise based CR which incorporate components of psychological treatment such as cognitive behavioural therapy (CBT), problem-solving therapy (PST), interpersonal psychotherapy (IPT) and psychodynamic therapy may address common symptoms of depression and anxiety experienced by MI survivors [25].

The cognitive responses to MI include the participants’ attitudes, beliefs and self-efficacy to PA. Generally, those with positive cognitive responses such as *“I’ve always been active”, “I just get on with it’, or “I can’t give up”* predicted participants ability to maintain or increase PA levels following MI. A study of patients with ischaemic heart disease (i.e. MI and angina) in Denmark found patients with positive attitudes were more likely to exercise and had a lower risk of mortality long term (5-year follow-up) as a result [26]. Similarly, among patients followed up at 4–10 days post-MI, 2 months post-MI and 8 months post-MI maintenance self-efficacy (i.e. a person’s baseline belief that they could sustain changes in PA long-term) predicted PA behaviour over follow-up, whereas recovery self-efficacy (i.e. a person’s belief they will recover from their MI) predicted relapse to sedentary habits [27]. Interventions to maximise long-term PA adherence could address different cognitions that predict attitude and self-belief [27, 28]. In practice this may involve different motivational techniques and resource recommendations dependent on baseline health beliefs and pre-MI awareness of the benefits of PA. Additionally, a focus on increasing positive attitudes towards PA is likely to improve adherence and engagement to CR programmes [26].

This study found considerable variation among participants experience of CR services across the UK [29]. Some participants described programmes involving structured group exercise and continuing support in the community whereas others discussed CR programmes provided on a one-to-one basis in a hospital setting. It is likely that format and delivery of CR influence engagement and participation. Variation in delivery has been cited as an explanation behind The National Audit for Cardiac Rehabilitation (NACR) statistics, which suggest UK CR participation rates range from 18 to 90 % [30]. Participants within this study also described a varied range of PA services offered following CR. These findings suggest a need to standardise follow on support once CR is completed so MI survivors have access to similar exercise classes and feel supported to maintain positive PA behaviours. We also found there is a need to ensure those who decline CR on the basis they exercise to a high level still receive guidance about how to safely return to pre-MI PA level. Resources provided by charitable bodies such as the BHF were praised for offering valuable information and peer support. Many asymptomatic participants wanted regular long-term contact with the cardiologist for reassurance; it is unclear practically how such needs could be met as in the UK secondary prevention care following MI (which includes PA) is overseen by the GP.

### Strengths and limitations

This study provides a qualitative insight into factors relating to PA behaviour post-MI. With the intention of creating a diverse and representative sample, a broad range of adults (gender, age, UK location) were recruited. Participants were heterogenous in their clinical condition, which provided a more comprehensive description of living post-MI. However, data on the co-morbidities of patients and the specific programme of CR they attended was not gathered. This data would have been informative about the representation of the sample to the wider post-MI population. Although telephone interviews were chosen for convenience, selection and response bias is likely. For example, most participants were recruited via cardiac related online platforms; it can be assumed that those who volunteered to take part are more interested and engaged in CR services, cardiovascular disease support and leading a healthy lifestyle. Within this study the sample was younger than typical post-MI patients thus limiting the generalisability of the results. Theoretical sampling whereby data collection and analysis occur simultaneously to guide subsequent recruitment could have been used [31]. The depth of the data presented could be further expanded by further recruitment and interviews, and by incorporating multiple methods of data collection such as focus groups which would have allowed for participant-led discussion about the variation in post-MI support and CR services. Nevertheless, the results of this study provide a preliminary depiction of some of the relevant factors for improving PA post-MI.

## Conclusions

Post-MI, once cardiac rehabilitation ends, affective and cognitive factors influence patients’ receptivity and motivation to maintain positive behaviour change. This study suggests interventions that improve heart attack survivors’ self-efficacy to exercise by addressing PA beliefs and perceived ability to be active may be beneficial. Providing ongoing PA advice and access to social support may facilitate heart attack survivors to maintain changes in PA acquired following their MI.

## Implications for clinical practice

- MI survivors’ affective emotional response to their condition (i.e. concern, fear and anxiety towards MI) and cognitive appraisal of PA (i.e. self-efficacy to exercise and attitude towards PA) influence their engagement with CR and maintenance of PA long-term.
- Many MI survivors describe a lack of available guidance on maintaining PA behaviour change following CR and that advice on the frequency and intensity of exercise to follow is often unclear and confusing.
- Interventions which improve MI survivors’ self-efficacy to exercise by addressing PA beliefs and perceived ability to be active may be beneficial.
- Providing ongoing PA advice and access to social support may facilitate MI survivors to maintain changes in PA acquired following their heart attack.

## Supplementary Information

**Additional file 1**. The COnsolidated criteria for REporting Qualitative research (COREQ).

## Data Availability

The datasets used and/or analysed during the current study are available from the corresponding author on reasonable request.

## Abbreviations

PA: Physical activity
CR: Cardiac rehabilitation
MI: Myocardial infarction

## References

[1] BHF. British Heart Foundation Statistics Factsheet, UK. 14/12/2018 (2018). https://www.bhf.org.uk/what-we-do/our-research/heart-statistics.

[2] Mampuya WM (2012). Cardiac rehabilitation past, present and future: an overview. Cardiovasc Diagn Ther.

[3] Crowther CA (2016). Mid-childhood outcomes of repeat antenatal corticosteroids: a randomized controlled trial. Pediatrics.

[4] The British Association for Cardiovascular Prevention and Rehabilitation (BACPR). Standards and core components for cardiovascular disease prevention and rehabilitation, 3rd ed. (2017) http://www.bacpr.com/resources/6A7_BACR_Standards_and_Core_Components_2017.pdf.

[5] Piepoli MF (2012). Secondary prevention in the clinical management of patients with cardiovascular diseases. Core components, standards and outcome measures for referral and delivery: a policy statement from the cardiac rehabilitation section of the European Association for Cardiovascular Prevention and Rehabilitation. Endorsed by the Committee for Practice Guidelines of the European Society of Cardiology. Eur J Prev Cardiol.

[6] Dibben GO (2018). Cardiac rehabilitation and physical activity: systematic review and meta-analysis. Heart.

[7] Racodon M, Pezé T, Masson P (2019). Analysis of physical exercise in cardiac patients following cardiovascular rehabilitation. Acta Cardiol.

[8] Blanchard CM (2014). Examining the steps-per-day trajectories of cardiac rehabilitation patients: a latent class growth analysis perspective. J Cardiopulm Rehabil Prev.

[9] Murray J (2013). A qualitative synthesis of factors influencing maintenance of lifestyle behaviour change in individuals with high cardiovascular risk. BMC Cardiovasc Disord.

[10] Ajzen I (1991). The theory of planned behavior. Organ Behav Hum Decis Process.

[11] Cassidy E (2011). Using interpretative phenomenological analysis to inform physiotherapy practice: an introduction with reference to the lived experience of cerebellar ataxia. Physiother Theor Pract.

[12] Glaser B, Strauss A (1967). The discovery of grounded theory: strategies for qualitative research.

[13] Fugard AJB, Potts HWW (2015). Supporting thinking on sample sizes for thematic analyses: a quantitative tool. Int J Soc Res Methodol.

[14] Braun V, Clarke V (2006). Using thematic analysis in psychology. Qual Res Psychol.

[15] Tierney S (2011). What influences physical activity in people with heart failure? A qualitative study. Int J Nurs Stud.

[16] Tofler GH (2015). Acute coronary syndrome as a teachable moment for smoking cessation. J Smok Cessat.

[17] McBride CM, Emmons KM, Lipkus IM (2003). Understanding the potential of teachable moments: the case of smoking cessation. Health Educ Res.

[18] Lawson PJ, Flocke SA (2009). Teachable moments for health behavior change: a concept analysis. Patient Educ Couns.

[19] Lazarus RS (1993). Coping theory and research: past, present, and future. Psychosom Med.

[20] Mills AL, Pierce JP (2008). Using teachable moments to improve nutrition and physical activity in patients. Am Fam Phys.

[21] Nicolai J (2018). To change or not to change—That is the question: a qualitative study of lifestyle changes following acute myocardial infarction. Chronic Illn.

[22] Ahlund K, Back M, Sernert N (2013). Fear-avoidance beliefs and cardiac rehabilitation in patients with first-time myocardial infarction. J Rehabil Med.

[23] Boehm JK, Kubzansky LD (2012). The heart’s content: the association between positive psychological well-being and cardiovascular health. Psychol Bull.

[24] Zheng X (2019). Effect of exercise-based cardiac rehabilitation on anxiety and depression in patients with myocardial infarction: a systematic review and meta-analysis. Heart Lung.

[25] Anderson L (2016). Exercise-based cardiac rehabilitation for coronary heart disease. Cochrane Syst Rev Meta Anal.

[26] Hoogwegt MT (2013). Exercise mediates the association between positive affect and 5-year mortality in patients with ischemic heart disease. Circ Cardiovasc Qual Outcomes.

[27] Luszczynska A, Sutton S (2006). Physical activity after cardiac rehabilitation: evidence that different types of self-efficacy are important in maintainers and relapsers. Rehabil Psychol.

[28] Scholz U, Sniehotta F, Schwarzer R (2005). Predicting physical exercise in cardiac rehabilitation: the role of phase-specific self-efficacy beliefs. Clin J Sport Med.

[29] NICE. Myocardial infarction: cardiac rehabilitation and prevention of further cardiovascular disease. Clinical guideline [CG172]. Published date: November; 2013. 31891465

[30] National Audit of Cardiac Rehabilitation (NACR) (2011). Annual statistical report 2011.

[31] Ruppel PS, Mey G (2017). Grounded theory methodology.

