# Supplementary material for: Maintaining physical activity following myocardial infarction: a qualitative study
Source: BMC Cardiovasc Disord. 2021 Feb 18;21:105. doi: 10.1186/s12872-021-01898-7 (PMC7893716; doi:10.1186/s12872-021-01898-7)
Supplement: Supplementary file 1 — Additional file 1. The COnsolidated criteria for REporting Qualitative research (COREQ). [file 12872_2021_1898_MOESM1_ESM.docx]

**Additional file A**

**Title**

‘A Qualitative Study of Physical Activity following Myocardial Infarction’

**Aims and Objectives**

To explore factors influencing the PA behaviour of post-MI patients including patients’ attitude and appraisal towards PA, and perceived barriers, motivators and facilitators for maintenance of PA longer term.

**General points**

- Semi-structured to facilitate natural flow of conversation
- Mainly open-questions allowing the participant to speak freely
- Prompts should be used to guide and encourage participants to elaborate
- Focus on participant’s perception of their health and physical activity
- Appraisal is defined as the cognitive evaluation and value judgement by an individual towards an event. In the context of physical activity it refers to how an individual views and values the health benefits of physical activity.

**Introductions & House-keeping**

- Introduce self and the aim of the interview.
- Check the length of the session is OK.
- Highlight fire exits and bathrooms if interview is face-to-face.
- Check the recording software is working, and that the participant can hear and see the interviewer if being conducted remotely via video chat.
- Remind the participant that the discussion is confidential and that their views and experiences will be presented and discussed in a non-identifiable manner.
- Remind the participant that if they wish to pause or end the discussion at any time, they are free to do so.
- Ensure the participant has completed the brief one-page questionnaire before the interview commences, which includes their initials, age, gender, cardiac diagnosis and management.

**Interview Questions**

1. Could you give me a brief **overview of what happened** when you had your heart attack? Prompts: what / when / treatment / recovery
2. What, if any, **physical activity** or exercise do you do **currently**? Prompts: when / where / with who / how long
3. How has your heart attack changed your **day-to-day lifestyle**? Prompts: ADLs / job / family life / what has been toughest / key impacts and changes
4. What **advice** did you receive about **physical activity** following a heart attack? Prompts: when / by whom (in-hospital from nurses or doctors / GP / friends or relatives been through similar) / who with (e.g. relative / friend) / when / in what form (informal, written, telephone, website etc)
5. What has been you **experience of following this advice** in the short and longer term? Prompts: what has been easy / more challenging / why
6. Is there any **advice or support** in relation to physical activity that you **would like to have received** but did not? Prompts: at what stage / how much / by whom / in what form / ongoing support
7. Describe your physical activity levels **before** vs **after** the heart attack vs **now**? Prompts: type / duration / time of day or week / group or individual / were they ‘active’ when younger / do they see themselves as active or sedentary
8. Tell me about what **has motivated** you to be more active / or what **would motivate** to be more active? Prompt: explore facilitators to PA
9. What **barriers** have you come across that stop you being more active? Prompts: time (return to work, family or other responsibilities) / lack of motivation / boredom / stress events / safety of outdoor spaces / lack of company / worry about further cardiac events / co-morbidities e.g. OA / cardiac symptoms e.g. angina
10. Do you know how much physical activity is **recommended** by the government? Prompt: per week 150 mins moderate intensity or 75 mins vigorous, 2 strength sessions / could they give some examples…
11. Tell me what you know about **cardiac rehabilitation programmes**? Prompt: structured exercise sessions, psychological support, education on topics such as medications and lifestyle modification recommendations
12. Did you **complete** a cardiac rehabilitation programme? Prompt: why did or did they not take up the offer / if dropped out, why
13. Do you do any **ongoing supervised physical activity** (such as a cardiac group / personal trainer / walking group etc)? Prompt: how have you managed to maintain being active?
14. If you wanted **advice and support** about physical activity where would you go, or who would you ask? Prompt: GP / cardiac nurses / cardiologist / support groups / fellow patients / relatives / friends / websites / forums / telephone lines
15. Is there **anything you would like to add** about your experience with physical activity and heart disease that we have not already spoken about? Prompt: anything they would like to reflect on / topics not mentioned / questions

**Post interview**

- Thank the participant for their time and stop the recording
- Collate any notes written down during the interview
- Reflect how the interview felt and flowed, including the participant’s non-verbal cues. Note any key thoughts, feelings or themes
- Ensure data is stored securely

**Additional file B**

**Questionnaire**

- To be completed prior to starting the interview
- Please fill in the blanks or circle the correct response as appropriate

**Title**: _______________

**First** **name**: _______________

**Surname**: _______________

**Age**: _______________

**Gender**: _______________

**Date** **of first heart attack**: ________ / ________ (month / year)

1. Do you have any significant hearing troubles that would prevent you partaking in the study? **Yes / No**
2. Do you suffer from impaired memory, or have a diagnosis of dementia, that means you struggle to recall information accurately? **Yes / No**
3. Do you suffer from any other illnesses that may be relevant to you taking part in the interview? **Yes / No**
   1. If yes, details: _____________________________________________
4. Is there anything you would like the investigators to be aware of before we start the interview? **Yes / No**
   1. If yes, details: _____________________________________________
